# Mediterranean PAH Stratification in Phenylketonuria: Tracing Historical Maps to Point Toward Clinical Phenotype and Obesity Risk

**DOI:** 10.3390/genes17080861

**Published:** 2026-07-24

**Authors:** Albina Tummolo, Ornella Tabaku, Eleonora Meneleo, Ciro Leonardo Pierri, Anna De Grassi, Vito Di Tullio, Giulia Paterno, Donatella De Giovanni

**Affiliations:** 1 Department of Inherited Metabolic Disorders and Clinical Genetics, Children Hospital Giovanni XXIII, Azienda Ospedaliero-Universitaria Consorziale, 70126 Bari, Italy; 2 Department of Pharmacy-Pharmaceutical Sciences, University of Bari “Aldo Moro”, Via E. Orabona 4, 70125 Bari, Italy; ciro.pierri@uniba.it; 3 Department of Biosciences, Biotechnologies and Environment, University of Bari “Aldo Moro”, Via E. Orabona 4, 70125 Bari, Italy

**Keywords:** phenylketonuria (PKU), hyperphenylalaninemia (HPA), *PAH* gene, population genetics, Mediterranean genetics, genotype-phenotype correlation, precision medicine, geographical clustering, metabolic risk, Body Mass Index (BMI), BH4 responsiveness, Pegvaliase, historical migrations, Southern Italy

## Abstract

Background: Apulia and neighbouring Southern Italian regions present a complex genetic stratification shaped by historical migrations. Phenylketonuria (PKU), caused by *PAH* mutations, serves as an excellent model for molecular epidemiology. This study maps the geographical distribution of *PAH* variants in a large Southern Italian cohort, evaluating their correlation with clinical phenotypes and long-term Body Mass Index (BMI). Methods: A retrospective cohort study was conducted on 344 patients followed for at least 10 years. Genetic characterization evolved from Sanger to Next-Generation Sequencing, and patients were stratified into micro-geographic clusters. Results: The genetic landscape was dominated by three ancestral variants (c.1208C>T, c.1066-11G>A, c.898G>T), accounting for 58% of the allelic pool. Significant micro-geographic polarization was observed: c.1222C>T clustered in Central–Northern Apulia, mirroring historical Norman-Swabian migration, whereas c.441+5G>T enriched in Southern Apulia. At final follow-up, classic PKU adult patients exhibited a four-fold increased risk of obesity (*p* < 0.05), driven by severe null alleles that necessitate a lifelong dependency on engineered low-protein foods. Conclusions: *PAH* mutational stratification serves as a contemporary reflection of historical migratory maps. Incorporating regional and genotypic mapping provides a precision medicine framework to anticipate phenotype severity, optimize therapeutic management, and tailor long-term obesity risk monitoring.

## 1. Introduction

Apulia, due to its peculiar geographical position as a link between the Mediterranean and continental Europe, has been the scene of complex demographic stratifications over millennia. The genetic history of the region reflects this geographic position and is the result of large migratory impulses. Beginning in the 8th century BC, Magna Graecia colonization established the first solid genetic links with the Levant basin [1]. This flow was consolidated in the following centuries by Byzantine domination, which maintained Bari and Salento as central nodes of a Mediterranean biological network [2]. Between the 11th and 13th centuries, Apulia underwent a radical political and demographic transformation with the arrival of the Normans and the Swabians. These groups, originating from Northern Europe and Normandy, introduced genetic variants typical of central–northern populations, settling in particular in the centres of Frederick’s power [3]. Parallel to the dynamic coasts, the inland areas of the Murgia and Apennines have experienced periods of relative isolation, promoting genetic drift phenomena and the conservation of native or rare variants [4].

In human genetics, Phenylketonuria (PKU; OMIM #261600) is considered one of the premier models for molecular epidemiology, as its mutations can act as true historical markers [5]. The European distribution of Phenylalanine Hydroxylase (*PAH*, OMIM 612349) gene variants is highly heterogeneous and follows precise geographic gradients [6]. Mutations such as p.Arg261Gln (Exon 7) show a high prevalence in the Mediterranean basin and Southeastern Europe. They are often associated with moderate or mild biochemical phenotypes, reflecting a metabolic adaptation typical of populations that have historically consumed a diverse Mediterranean diet [7]. The c.1222C>T (p.Arg408Trp) mutation (Exon 12) is the most common variant globally, with a peak prevalence in Eastern Europe as well as in Germanic and Scandinavian countries. This variant is almost always associated with severe classical PKU, with zero enzymatic activity. Its presence in Southern Italy is historically traceable to medieval migratory flows [8]. Other mutations, such as IVS10nt-11 or c.1162G>A (p.Val388Met), are characteristic of the Mediterranean area (Italy, Spain, North Africa), proving a genetic continuity among populations bordering the same sea [9].

Since historically severe PKU did not allow affected individuals from reproducing (prior to the introduction of the life-saving dietary therapy in the 1960s) [10], the persistence of these mutations has been kept exclusively through healthy carriers [11]. In the absence of strong negative selective pressures against healthy carriers, frequency of the mutation remained stable, acting as a genetic signature of ancient populations until the present day [12]. While the mutational spectrum of *PAH* deficiency is well documented, the correlation between geographical clustering, gender–age distribution, therapeutic modalities [13,14,15,16] and long-term anthropometric outcomes such as Body Mass Index (BMI) remains underexplored in the context of precision medicine [17,18]. The hypothesis underlying this work is that the geographical origin of PKU patients, precisely tracing historical maps, can predict specific type of PKU phenotypes, guide therapeutic choices, and ease targeted interventions for long-term obesity risk management.

## 2. Materials and Methods

### 2.1. Study Design and Population

A retrospective study was conducted on subjects affected by *PAH* deficiency, followed-up at the Reference Center for Metabolic Diseases of the Apulia Region. Patient data from the initial evaluation were entered into a database using pseudo anonymized identifiers. Information regarding outpatient visits and hospitalizations has been entered. Longitudinal data regarding outpatient visits and hospitalizations were systematically recorded. Exclusion criteria were represented by HPA due to BH4 deficiency and loss-of-follow-up in the last 5 years. Patients followed up for a study period of more than 10 years (2009–2026) were included. The population was categorized into three main groups considering the phenotypic classification based on diagnostic, pre-treatment blood phenylalanine (Phe) levels, following the European PKU guidelines [15]. Classic PKU group: patients with Phenylalanine (Phe) levels at birth > 1200 umol/L, patients with mild–moderate PKU: Phe values > 600 and <1200 umol/L, both groups requiring treatment; Hyperphenylalaninemia (HPA) group: patients with Phe levels at birth < 360 umol/L and natural protein tolerance not requiring therapeutic intervention. Pre-treatment biochemical classification was established using quantitative blood amino acid analysis.

Data collection was conducted in full compliance with the national newborn screening legal framework; formal ethical review was waived under Italian Law No. 167/2016 and Apulia Regional Resolution No. 331/2018, as detailed in the Institutional Review Board Statement.

### 2.2. Genetic Characterization and Geographical Stratification

For each patient, the *PAH* genotype was characterized. The molecular analysis was conducted following the evolution of the sequencing technologies available over time. For historically samples, genotype determination was performed by direct sequencing using the Sanger method, focusing the investigation on the coding regions (the 13 exons) and their relative splicing junctions. During the initial phase of the study, standard molecular screening and genotype determinations were performed using traditional Sanger sequencing on Applied Biosystems platforms (Thermo Fisher Scientific, Waltham, MA, USA). This capillary electrophoresis-based methodology served as the primary diagnostic tool for patients recruited during the earlier years of the follow-up period.

Subsequently, advanced genetic screening shifted to Next-Generation Sequencing (NGS) architectures to enable massive parallel sequencing of millions of DNA fragments simultaneously. Clinical Exome Sequencing (CES) was performed on the Illumina NextSeq550Dx platform (Illumina, San Diego, CA, USA) using the TruSight One Sequencing Panel.

This approach enabled a comprehensive analysis of the entire gene locus, extending the search beyond the exonic sequences. In particular, the NGS workflow included the systematic evaluation of deep intronic variants, to identify mutations that could alter splicing or transcript stability, which would otherwise be undetectable with traditional sequencing methods [19]. The classification of the identified variants was performed in accordance with international guidelines (ACMG/AMP) [20].

Patients were geographically stratified based on their municipality of birth/residence to identify territorial clusters: Northern–Central Apulia (municipalities of the metropolitan area of Bari and the Adriatic coast, Area of Barletta, Andria, Trani and neighbouring municipalities); Southern Apulia (municipalities of Taranto, Brindisi and Lecce); Extra-Regional Cluster (subjects coming from other Italian Regions); Foreign Cluster (subjects with foreign parents). The classification into Northern–Central Apulia (Bari, BAT, Foggia) and Southern Apulia (Taranto, Brindisi, Lecce) was chosen to reflect well-documented historical–linguistic boundaries and genetic gradients (the Salento peninsula vs. the continental north–central plains). Basilicata was kept separate due to its distinct mountainous isolation. To minimize misclassification bias, patients were assigned to a regional cluster only if their documented municipality of birth and long-term residence (spanning the 10-year follow-up) aligned within the same geographical macro-area. Individuals with discordant birth and residence records or highly transient migration histories within the region were excluded from the micro-geographic sub-analyses.

### 2.3. Anthropometric Assessment

Anthropometric parameters, specifically weight and height, were systematically measured by trained clinical staff during routine follow-up outpatient visits. Weight was recorded to the nearest 0.1 kg using a calibrated electronic medical scale (Baby One—Wunder; Seca Vogel & Haike Hamburg 715, Hamburg, Germany), with patients dressed in light clothing and barefoot. Standing height was measured to the nearest 0.1 cm using a wall-mounted stadiometer (Seca 417 Hammer Steindamm, Hamburg, Germany; Seca Vogel & Haike Hamburg 715), with patients positioned according to standard anthropometric protocols (heels together, shoulders relaxed, and head aligned in the Frankfurt horizontal plane). Body Mass Index (BMI) was subsequently calculated using the standard formula: BMI = weight (Kg)/height (m^2^). For the pediatric sub-cohort (defined as patients aged ≤ 18 years at the last follow-up, *n* = 219), individual BMI values were converted into sex- and age-specific percentiles in accordance with World Health Organization (WHO) growth standards. Overweight was defined as a BMI-for-age > 85th percentile and obesity as >97th percentile). For adult patients (aged > 18 years), overweight and obesity were categorized using standard absolute BMI thresholds of >25 kg/m^2^ and >30 kg/m^2^, respectively. To ensure updated metabolic and nutritional profiles, the most recent anthropometric data available at the final documented follow-up visit were used for the statistical analysis.

### 2.4. Therapeutic Management of the Cohort

To evaluate the clinical impact and genotype–phenotype correlation, the following therapeutic data were extracted: dietary therapy, dietary therapy combined with pharmacotherapy (BH4), and enzyme therapy. Therapeutic strategies applied to the study population were established in line with international guidelines for the management of PKU [15]. Dietary therapy with restricted Phe intake, associated with the administration of Phe-free amino acid mixtures, represented the first-line therapeutic approach. This was promptly started in all patients following a positive diagnosis via newborn screening or upon detection of persistent hyperphenylalaninemia requiring treatment (>360 umol/L) [21]. To assess the potential for partial or total liberalization of dietary Phe tolerance, patients underwent Tetrahydrobiopterin (BH4) responsiveness testing (via Sapropterin Dihydrochloride administration). BH4 therapy was initiated and maintained exclusively in subjects who demonstrated a significant biochemical response, defined as a persistent reduction in plasma Phe levels of 30% or greater relative to baseline values, or as a documented increase in dietary Phe tolerance [22,23]. The transition to enzyme substitution therapy with Pegvaliase was reserved for a selected subpopulation of adolescents and adult patients (aged ≥ 16 years). The inclusion criteria for initiating Pegvaliase required the presence of chronic inadequate metabolic control, defined as plasma Phe levels consistently above the therapeutic target of 600 umol/L, due to non-adherence to traditional dietary therapy, in subjects previously identified as non-responsive or refractory to BH4 treatment [24,25].

### 2.5. Statistical Analysis

Data were analyzed using the SPSS statistical software (version 17). The distribution of continuous variables was assessed using the Shapiro–Wilk test. For the comparison between two groups, Student’s *t*-test for independent samples (for normally distributed variables) or the Mann–Whitney U test (for non-parametric variables) was used. For the comparison between multiple groups (PKU types, geographical clusters), one-way Analysis of Variance (ANOVA) followed by Tukey’s post hoc test was performed for normally distributed data, while the Kruskal–Wallis test followed by Dunn’s post hoc test was used for non-parametric data. To control for family-wise Type I error inflation, post hoc pairwise adjustments (Tukey’s or Dunn–Bonferroni corrections) were strictly applied. Conversely, exploratory single-allele and micro-geographic association analyses were evaluated using raw *p*-values and 95% CI without global multi-testing adjustments, to preserve statistical power for rare genetic variants and avoid Type II error inflation; these exploratory findings should therefore be interpreted within this limitation. The prevalence of mutations across the different clusters was analyzed using the Chi-squared (chi^2^) test or Fisher’s exact test. The level of statistical significance was set at *p* < 0.05. The Odds Ratio (OR) was used to calculate the probability of presenting a specific PKU phenotype based on the alignment with a specific geographical cluster. To evaluate the impact of the genetic mutations on BMI, a logistic regression analysis was performed to determine the OR for elevated BMI (≥30 kg/m^2^, ≥97° percentile) across specific mutated alleles and phenotypic groups. To evaluate the impact of individual genetic mutations and clinical phenotypes on obesity risk, univariate (simple binomial) logistic regression analyses were performed to calculate the Odds Ratios (ORs) with 95% CIs. To avoid severe multicollinearity and overadjustment bias, alleles and clinical phenotypes were analyzed in separate, independent models. Furthermore, potential confounding by age and sex was addressed via strict cohort stratification: the pediatric sub-cohort (N = 216) was modelled using age- and sex-adjusted WHO BMI percentiles, while the adult sub-cohort (N = 103) was analyzed independently using absolute BMI thresholds.

## 3. Results

### 3.1. Demographic and Clinical Data

The overall cohort included a total of 344 patients affected by HPA and PKU. A female predominance was observed, with 190 patients (55%) compared to 154 males (45%). The mean age of the study population was 17.3 ± 12.2 years, with a range between 0.4 and 61.1 years. Mild HPA was the most frequent phenotype, identified in 179 subjects (52%), followed by classic PKU (92 patients, 26.7%) and mild–moderate PKU (73 patients, 21.2%). Concerning geographical origin, the vast majority of the analyzed cohort resided in the Apulia region (290 patients, 87.6%), with a main concentration in the provinces of Bari (95 patients, 32.7%) and Foggia (67 patients, 23.1%), followed by Taranto (47 patients, 16.2%), Lecce (46 patients, 15.8%), Barletta-Andria-Trani (BAT: 22 patients, 7.5%), and Brindisi (13 patients, 4.4%). A significant portion of the cohort comes from the neighbouring Basilicata region (38 patients, 11.4%, of whom 24 are in the province of Potenza and 14 in that of Matera), while the remaining 3 patients (0.9%) resided in the Calabria region (2 in Reggio Calabria and 1 in Cosenza). Thirteen patients were born to foreign parents (3.7%), all residing in the Apulia region, mostly from Albania and Romania (Table 1).

The comparative analysis of the demographic and therapeutic characteristics of the cohort, stratified according to the main clinical phenotype, highlighted statistically significant variations for almost all the variables considered (Table 2). First, the current age of the patients showed a marked asymmetry between the subgroups (*p* < 0.001): subjects affected by classical PKU presented a higher mean age (26.0 ± 15.3 years) compared to patients with HPA (13.1 ± 9.1 years) or mild PKU (15.9 ± 8.0 years), likely reflecting the historical diagnostic trends and differing eras of clinical interception within the territory. Gender distribution also revealed a significant heterogeneity (*p* = 0.010): in the HPA group and the most severe PKU phenotype, there is a clear prevalence of the female sex (61.4%, 38.5% respectively) while the mild PKU phenotype showed a specular trend, with a majority of male subjects (59.5%). From a geographical perspective, although the vast majority of the cohort resided permanently in the Puglia region (84.3%), the percentage of patients coming from outside the region or from abroad varied significantly depending on the phenotype (*p* = 0.029), reaching its peak in the mild PKU subgroup (23.2%) and its minimum in the HPA phenotype (10.6%). Conversely, the ethnic composition of the cohort was largely homogeneous (*p* > 0.05), with an almost total prevalence of the Italian component (96.2%) across all phenotypic groups. Finally, the analysis of the therapeutic profile confirmed, with an extremely high statistical significance (*p* < 0.001), the close and expected adherence between clinical phenotype and care strategy: the low-protein dietary therapy represented the cornerstone standard for classic PKU (88%). Conversely, Sapropterin use was predominantly concentrated in the mild PKU form (26%), and the advanced therapeutic approach with Pegvaliase was strictly limited to a selected proportion of adult patients with classical PKU (8.6%).

At the final follow-up visit, complete anthropometric data were available for 319 out of the 344 patients (93%). Given the wide age range of the cohort, nutritional assessments were stratified by age: adult patients (>18 years, *n* = 103) were evaluated using absolute BMI (kg/m^2^), while pediatric patients (≤18 years, *n* = 216) were assessed using BMI percentiles. Among adult patients, the overall mean BMI was 25.73 ± 5.06 kg/m^2^. Although the global comparison of BMI across the three clinical phenotypes did not reach statistical significance (*p* = 0.08, Kruskal–Wallis test), a progressive upward trend in BMI was observed as the phenotype severity increased, with patients in the HPA cohort exhibiting the lowest values (24.13 ± 4.39 kg/m^2^) while those in the Classical PKU group reached the highest (26.29 ± 4.43 kg/m^2^). Post hoc pairwise comparisons confirmed a statistically significant difference specifically between the HPA and Classical PKU adult subgroups (*p* < 0.05). In the pediatric sub-cohort, the overall mean BMI percentile was 68.18 ± 30.89. Similarly to the adults, pediatric patients with Classical PKU showed the highest average percentile (73.29 ± 29.39), followed by the Mild PKU (70.35 ± 31.85) and HPA (65.57 ± 31.44) groups, though these differences did not reach statistical significance (*p* = 0.22, Kruskal–Wallis test).

### 3.2. Allele Frequency and Distribution

Out of the more than 500 alleles identified, the three most frequent mutations, specifically the missense variant c.1208C>T (p.Ala403Val), the splicing mutation c.1066-11G>A, and the missense c.898G>T (p.Ala300Ser), alone account for a preponderant share of the total allelic pool in the study population (58%). Table 3 reports the distribution and frequency of major allelic variants by mutation type, historical/anthropological flow, and geographic origin. The genetic landscape is dominated by a solid indigenous and Magna Grecia substrate linked to the central–eastern Mediterranean basin. This is primarily represented by the mild missense mutation c.1208C>T (p.Ala403Val), which was the most frequent overall with 92 occurrences distributed between Central–Northern (46.7%) and Southern Apulia (37.0%), and by the c.143T>C (p.Leu48Ser) variant with 27 cases. Alongside this native background are genetic variants linked to ancient Middle Eastern and Byzantine routes, such as the c.1066-11G>A null splicing mutation (60 total cases, 51.7% of which are concentrated in Central–Northern Apulia), as well-established, sedentary Central–Western European lineages, such as the c.781C>T (p.Arg262Ter) null nonsense variant, detected 31 times with a high concentration in the province of Bari. Furthermore, ancient Neolithic and Hellenistic migratory routes connecting the Balkans and Asia Minor to Southern Europe are reflected in the moderate-to-severe c.898G>T (p.Ala300Ser) variant, identified 48 times and equally distributed mainly between Bari and Foggia.

The Slavic variant c.1222C>T (p.Arg408Trp) stands as a clear testament to medieval Norman and Swabian rule, showing a strong geographical polarization across north–central Apulia (81.2% of the 16 total cases, concentrated across the Bari, BAT, and Foggia areas) and a sharp contrast to southern-weighted variants such as c.441+5G>T, which is predominantly found in the Salento peninsula (62.5%).

### 3.3. Geographical Allocation and Phenotypic Risk of PAH Gene Variants

The analysis of the Apulian macro-areas revealed that testing Central–Northern Apulia against all other regions combined, the odds of identifying the c.1222C>T Exon 12 mutation were 4 times higher in patients from Central–Northern Apulia compared to the rest of the examined territory OR = 4.00, 95% CI: 1.13–14.22), and this result is statistically significant (*p* = 0.0226) with a confidence interval that excludes 1, thereby confirming that this geographic clustering is not a random fluctuation. A highly significant geographical polarization was also observed for the severe splice-site mutation c.441+5G>T. Individuals residing in Southern Apulia (Taranto, Lecce, and Brindisi) exhibited a more than four-fold increased odds of harbouring this specific variant compared to those from Central–Northern Apulia OR = 4.58, 95% CI: 1.40–15.00, *p* = 0.0094). Regarding the allelic variant p.Arg262Ter, this nonsense mutation showed a distinct over-regional enrichment trend in the Basilicata region (Potenza and Matera). The allelic frequency is nearly doubled compared to the rest of the cohort OR = 1.93, 95% CI: 0.76–4.90, *p* = 0.1540), defining a localized Apennine enclave although not reaching a statistical significance.

**Figure 1:**
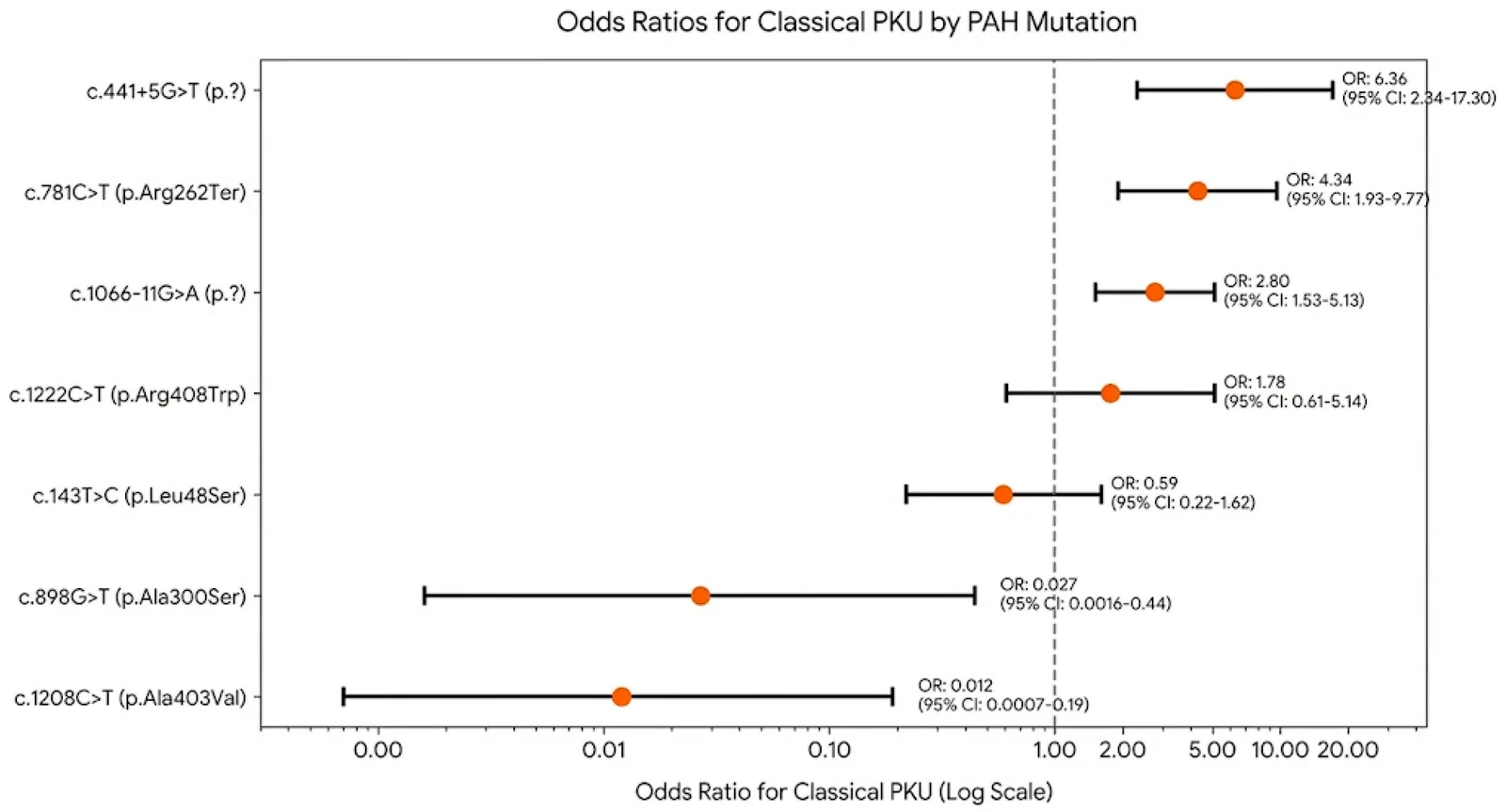
Association between individual mutated *PAH* alleles and the clinical presentation of Classical PKU (*n* = 330). Odds Ratios (OR) and 95% Confidence Intervals (CI) are unadjusted and calculated via univariate logistic regression. The reference group for each allele/phenotype consists of all other patients in the cohort not carrying that specific allele or phenotype.

Figure 1 illustrates the association between individual mutated *PAH* alleles and the clinical presentation of Classical PKU within our cohort.

The reference group for each individual allele/phenotype consists of all other patients in the cohort (N = 330) not carrying that specific allele or clinical phenotype. In line with the autosomal recessive nature of PAH deficiency, where the clinical phenotype is strictly determined by the milder of the two co-inherited mutations, these single-allele associations capture the distribution of genotypic combinations in our population. Specifically, the severe splice-site variant c.441+5G>T (OR = 6.36, 95% CI: 2.34–17.3, *p* = 0.0002) and the nonsense mutation c.781C>T (p.Arg262Ter) (OR = 4.34, 95% CI: 1.93–9.77, *p* = 0.0005) show the strongest statistical association with the classical form. This high association reflects the high probability in our cohort of these severe alleles being inherited either in homozygosity or in combination with other severe null variants, without being clinically masked by a co-inherited mild allele. The Mediterranean c.1066-11G>A variant also marks a significantly elevated risk OR = 2.80; 95% CI: 1.53–5.13; *p* = 0.0012). Conversely, the severe missense mutation c.1222C>T (p.Arg408Trp), despite showing a positive risk trend (OR = 1.78), does not reach independent statistical significance at the single-allele level (*p* = 0.3750), capturing the masking effect of mild variants in compound heterozygous states. Finally, the mild missense variant c.143T>C (p.Leu48Ser) plots to the left of the reference line (OR = 0.59), confirming its protective trend and association with milder hyperphenylalaninemia or Mild PKU phenotypes.

**Figure 2:**
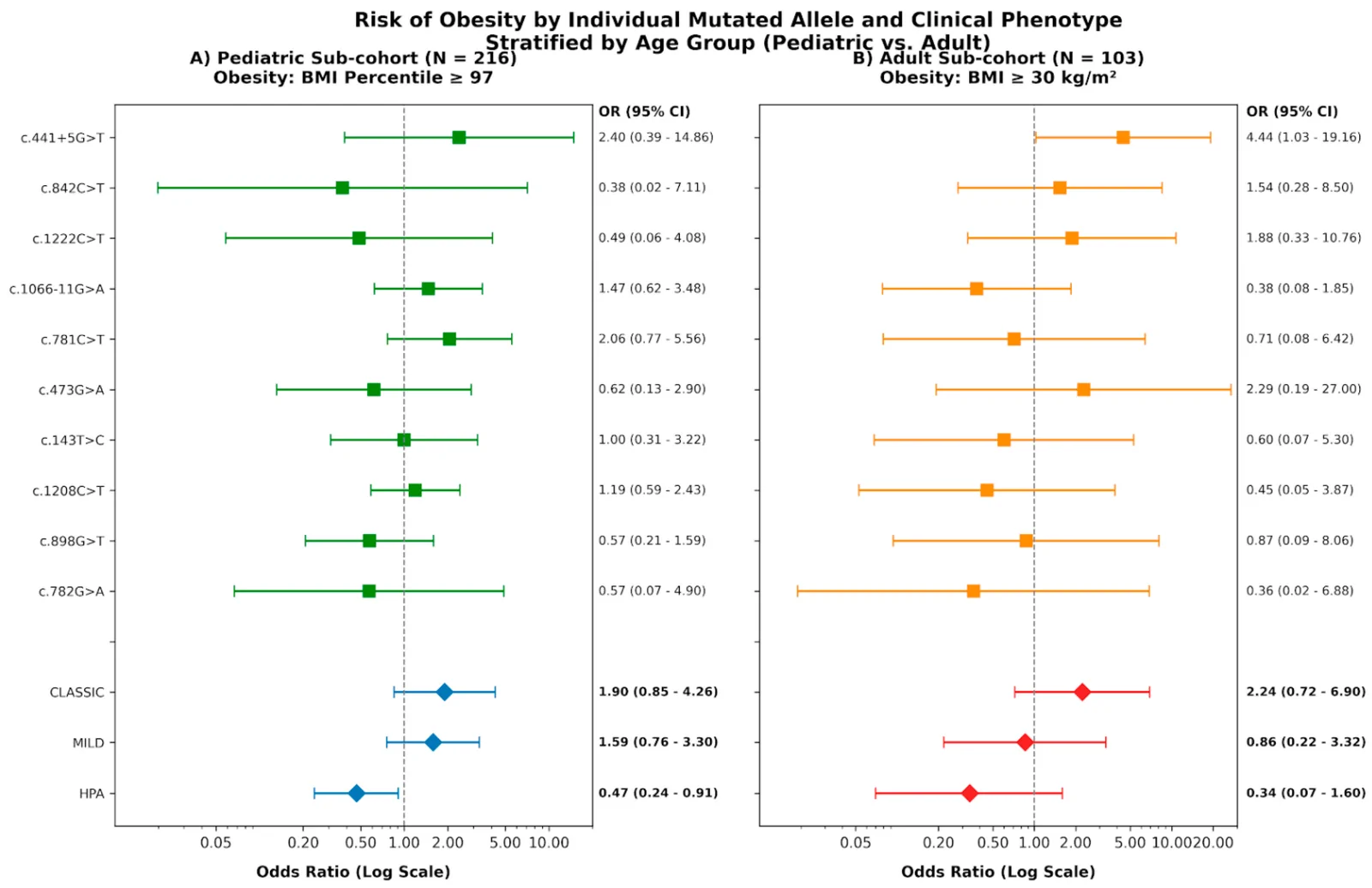
Risk of obesity by individual mutated allele and clinical phenotype, stratified by age group: (**A**) pediatric sub-cohort (*n* = 216, obesity defined as BMI percentile ≥ 97) and (**B**) adult sub-cohort (*n* = 103, obesity defined as BMI ≥ 30 (kg/m^2^). Odds Ratios (OR) and 95% Confidence Intervals (CI) are unadjusted and calculated via univariate logistic regression. The reference group for each estimate consists of all other patients within the respective stratified sub-cohort not carrying that specific allele or phenotype.

Stratification of the risk of obesity by single-allele detection is reported in Figure 2A,B and distinguished by age. The reference group consists of all other patients within the respective stratified age group (pediatric or adult) who do not present the target allele or clinical phenotype. In the pediatric subgroup (*n* = 216), patients with a mild HPA phenotype demonstrated a highly significant, lower risk of developing pediatric obesity (defined as BMI percentile > 97° according to WHO standards) with an Odds Ratio (OR) of 0.47 (95% CI: 0.24–0.91, *p* < 0.05). Conversely, pediatric patients with Classical PKU exhibited a clear trend toward an increased risk of obesity (OR = 1.90, 95% CI: 0.85–4.26), although this did not reach independent statistical significance. Among single-allele associations in children, the severe nonsense mutation c.781C>T (p.Arg262Ter) showed a pronounced trend toward pediatric adiposity (OR = 2.06, 95% CI: 0.77–5.56). In the adult subgroup (N = 103), where obesity was defined as BMI ≥30kg/m^2^, the phenotypic risk gradient became even more pronounced. Adults with Classical PKU presented a more than doubled risk of obesity compared to the rest of the adult cohort (OR = 2.24, 95% CI: 0.72–6.90). Most notably, the adult single-allele analysis revealed a highly significant, strong association between the regional splice-site mutation c.441+5G>T and adult obesity. Carriers of this severe null variant displayed a more than four-fold increased risk of developing obesity in adulthood (OR = 4.44 95% CI: 1.03–19.16, *p* < 0.05).

## 4. Discussion

This cohort study on patients with HPA and PKU provided a complex clinical and epidemiological mosaic in which wide phenotypic heterogeneity is dominated by HPA (52%), particularly frequent in female patients, and by a robust local recurrence effect of the ancestral Mediterranean variants c.1208C>T (p.Ala403Val) and c.1066-11G>A, yet interspersed with historical migratory trajectories that are highly polarized micro-geographically [23].

This is clearly evidenced by the relative enrichment of the c.781C>T variant in Basilicata (19.4%), the sharp segregation of the severe Norman-Swabian c.1222C>T (p.Arg408Trp) mutation in Central–Northern Apulia (81.2%), and the more frequent severe splice-site mutation c.441+5G>T in south Apulia (62.5%) [1,24]. Beyond tracing broad global movements, these ancestral lineages establish localized, over-regional enclaves in Southern Italy that dictate distinct contemporary clinical, metabolic, and dietary phenotypes [25].

In patients carrying the severe Norman-Swabian lineage (p.Arg408Trp), which forms a distinct micro-geographic hotspot in Central–Northern Apulia, total structural damage results in near-zero residual enzyme activity [11]. This enzymatic absence leads to a highly rigid clinical phenotype with minimal daily Phe tolerance (less than 250 mg/day) and a pronounced tyrosine deficiency that historically correlates with the fair skin, blonde hair, and light phototypes of northern European populations. Conversely, the Greco-Byzantine and Mediterranean ancestral substrate, characterized by the p.Arg261Gln variant common across Bari, Foggia, and Calabria, induces a partial, conformational instability rather than outright protein destruction [26]. This allows the mutant enzyme to retain 10% to 30% residual functional capacity, granting patients higher natural protein tolerance (500 to 800 mg/day) and a significant clinical responsiveness to Tetrahydrobiopterin therapy, which acts as a chemical chaperone to stabilize the protein [27,28].

When evaluating the phenotypic risk of individual variants, it is crucial to emphasize that *PAH* gene mutations do not act as independent risk factors; rather, the final clinical phenotype is dictated by the milder of the two inherited alleles [29]. Consequently, highly prevalent null alleles in our Southern Italian cohort, such as the Mediterranean c.1066-11G>A or the Norman-Swabian c.1222C>T, result in a classical PKU phenotype only when patients inherit two severe copies (homozygosity or severe compound heterozygosity). Conversely, when these null alleles are paired with highly prevalent mild missense variants, such as the indigenous c.1208C>T (p.Ala403Val), their severe biochemical impact is masked, resulting in milder hyperphenylalaninemia or mild PKU phenotypes [30]. The elevated Odds Ratios observed for certain severe variants in our single-allele analysis are therefore a direct consequence of their high allelic frequency and the relative lack of pairing with protective mild alleles within specific regional micro-geographic clusters.

The evolutionary persistence of severe, recessive *PAH* mutations within the Mediterranean gene pool may be explained by the heterozygote advantage hypothesis, which posits that carrying a single PKU allele offered ancestral survival benefits, such as protection against endemic mycotic toxins or infectious diseases [31,32,33,34,35]. Over centuries of dietary famine, this genetic background co-evolved with energy-thrifty genes (e.g., TCF7L2, FTO, PPARG, APOE, MC4R) to optimize lipid storage and metabolic efficiency [34,35,36,37]. In the modern era of nutritional abundance, this thriftiness triggers a severe evolutionary mismatch [38]. Specifically, for patients requiring lifelong restriction, the standard low-protein or protein-free PKU diet, characterized by a high glycaemic and lipid load, likely accelerates the development of insulin resistance and obesity [39,40]. This hypothesis is strongly supported by our clinical findings, which reveal a significant association between clinical phenotype severity and long-term obesity risk.

The results of this study reveal a strong, statistically significant association between the severity of the clinical phenotype and the risk of presenting with an elevated BMI in our patient cohort. In children, the most striking finding is the statistically significant protective effect of the Mild HPA phenotype against obesity (OR = 0.47, 95%CI: 0.24–0.91). This protective trend may be highly consistent with their natural, unrestricted diet, which avoids the high glycaemic and lipid loads of synthetic PKU medical foods [39]. Conversely, the adult sub-cohort highlights the long-term, cumulative metabolic toll of severe genotypes. Adult patients with Classic PKU carry a more than doubled risk of clinical obesity (OR = 2.24). Crucially, this age-stratified approach unmasked a highly significant association between the regional splice-site mutation c.441+5G>T and adult obesity, with adult carriers displaying a four-fold increased risk (OR = 4.44, 95% CI: 1.03–19.16, *p* < 0.05) that was undetected in children.

It is likely that this pronounced genotype-driven trend toward increased adiposity in adulthood is linked to a cumulative, time-dependent effect of lifelong adherence to highly engineered, carbohydrate-heavy low-protein products [17,38,41]. In severe genotypes (such as c.441+5G>T homozygotes), the complete absence of residual PAH activity enforces a strict, lifelong dependency on these medical foods to meet daily energy requirements.

Although this study provides valuable insights into the genetic and metabolic landscape of *PAH* deficiency in Southern Italy, several limitations must be addressed. First, the retrospective design spans a multi-decade follow-up period during which diagnostic molecular methodologies transitioned from traditional Sanger sequencing to high-throughput NGS, introducing an inherent historical variance in diagnostic depth. Second, while a significant genotype-driven risk for obesity was identified, our statistical models could not control for potential confounding variables such as socioeconomic status, baseline physical activity levels, or precise long-term dietary adherence rates. Furthermore, by relying on cross-sectional final endpoints (the most recent anthropometric data available at the last follow-up) rather than modelling longitudinal BMI trajectories, we were unable to map precisely when and how metabolic drift accelerates in these patients. Consequently, the identified metabolic associations should be interpreted as highly plausible hypotheses rather than definitive causal conclusions. Third, while our total cohort (N = 344) is robust for a rare metabolic disease, the data originate from a single regional reference centre. The observed geographic polarization of specific variants reflects localized founder effects that may not be directly generalizable to other global populations. Nevertheless, these findings closely align with comparable micro-geographic clustering and genotype–phenotype patterns recently reported in neighbouring Mediterranean areas, such as Eastern Sicily [40] and Turkey [41], reinforcing the clinical and epidemiological value of localized mutational mapping. We also acknowledge the potential for Type I error inflation due to multiple parallel hypothesis testing across various alleles, regions, and phenotypes. While primary comparisons for clinical classifications and BMI trajectories were protected by strict post hoc adjustments (Tukey and Dunn–Bonferroni corrections), single-allele and micro-geographic analyses were evaluated using raw *p*-values and 95% CI without global multi-testing corrections. In rare diseases characterized by highly unequal allelic distributions, applying overly conservative corrections (such as the standard Bonferroni method) severely inflates Type II errors, potentially masking clinically relevant localized trends. Therefore, these single-allele associations must be interpreted as exploratory, hypothesis-generating observations requiring prospective validation in larger, independent multicenter cohorts. Finally, it is crucial to clearly distinguish between our evidence-based clinical observations and the historical–anthropological interpretations regarding the ancestral origins of these PAH variants. While the modern micro-geographic clustering of specific alleles within Apulia is objectively demonstrated by our data, the proposed correlations with ancestral migrations, such as the Norman-Swabian, Byzantine, or Magna Graecia historical flows, remain speculative. Because this study did not include formal population genetics analyses, such as haplotype conservation, admixture patterns, or founder effect modelling, these historical links represent plausible literature-based interpretations supported by external molecular anthropology rather than evidence-based findings [1,2,3,4,11,26]. Consequently, while these historical perspectives enrich our understanding of regional epidemiology, contemporary clinical management must always be guided strictly by patient-specific clinical parameters, phenotypic severity, and genetic results rather than historical geographic ancestry.

## 5. Conclusions

Studying the geographical origin of a mutation is a valuable clinical tool which may help clinicians anticipate disease severity and understand the clinical behaviour of the mutation. When integrated into a comprehensive, multi-dimensional assessment of patient-specific clinical, nutritional, and lifestyle factors, it can help clinicians identify high-risk subgroups early and design more personalized metabolic follow-ups focused on monitoring BMI trajectories and mitigating obesity risks.

## Figures and Tables

**Table 1:** Population’s demographic characteristics.

	Frequency (*n*)	Percentage (%)
Sex (*n* = 344)		
Female	190	55%
Male	154	45%
Age (years)		
Mean ± Standard Deviation	17.3 ± 12.2	
Range	0.4–61.1	
Clinical Classification (Phenotype) (*n* = 344)		
Mild Hyperphenylalaninemia (HPA)	179	52%
Classical Phenylketonuria (PKU)	92	26.7%
Mild Phenylketonuria (PKU)	73	21.2%
BMI (*n* = 319)		
Mean ±Standard Deviation	21.13 ± 5.52	
Geographical Origin by Region (*n* = 331)		
Apulia	290	87.6%
Basilicata	38	11.4%
Calabria	3	0.9%
Distribution across Apulian Provinces (*n* = 290)		
Bari (BA)	95	32.7%
Foggia (FG)	67	23.1%
Taranto (TA)	47	16.2%
Lecce (LE)	46	15.8%
Barletta-Andria-Trani (BAT)	22	7.5%
Brindisi (BR)	13	4.4%
Parents’ Nationality (*n* = 344)		
Born to foreign parents (residing in Apulia)	13	3.7%
Albania	5	
Romania	3	
Marocco	2	
China, Armenia, Tunisia	3	

**Table 2:** Patient characteristics according to clinical phenotypes.

Item/Characteristic	Total Cohort (*n* = 344)	HPA (*n* = 179)	Mild PKU (*n* = 73)	Classical PKU (*n* = 92)	*p*-Value
Age (years), Mean ± SD	17.3 ± 12.2	13.1 ± 9.1	15.9 ± 8.0	26.0 ± 15.3	<0.001
Sex, *n* (%)					0.010
Females	189 (55%)	110 (61.4%)	30 (40.5%)	50 (54.3%)	
Males	154 (45%)	69 (38.5%)	43 (59.5%)	42 (45.7%)	
Origin, *n* (%)					0.029
Apulia Region	2903 (84.3%)	160 (48.3%)	56 (76.7%)	74 (80.4%)	
Out of Region/Abroad	54 15.6%)	19 (10.6%)	17 (23.2%)	18 (19.5%)	
Type of Therapy, *n* (%)					<0.001
None	217 (63%)	179 (100%)	38 (52%)	0	
Low-protein diet	97 (28.1%)	0 (0%)	16 (21.9%)	81 (88%)	
Sapropterin	22 (6.3%)	0 (0%)	19 (26.0%)	3 (3.2%)	
Pegvaliase	8 (2.3%)	0 (0%)	0 (0.0%)	8 (8.6%)	
Nutritional Assessment	(*n* = 319)	(*n* = 154)	(*n* = 73)	(*n* = 92)	
BMI (kg/m^2^), Mean ± SD (>18 y)	25.73 ± 5.06	24.13 ± 4.39	25.52 ± 6.56	26.29 ± 4.43	0.08
BMI percentile, Mean ± SD (≤18 y)	68.18 ± 30.89	65.57 ± 31.44	70.35 ± 31.85	73.29 ± 29.39	0.22

**Table 3:** Distribution and frequency of major allelic variants by mutation type, historical/anthropological flow, and geographic origin.

Allelic Variant	Mutation Type	Historical/Anthropological Flow	Central–Northern Apulia(BA, BAT, FG)	Southern Apulia(TA, LE, BR)	Basilicata(PZ, MT)	Other/Not Spec.	Total Frequency
c.1208C>T (p.Ala403Val)	Missense (Mild)	Indigenous/Magna Grecia [8,24]	43 (46.7%)	34 (37.0%)	7 (7.6%*)*	8 (8.7%)	92
c.1066-11G>A (p.?)	Splicing (Null)	Middle Eastern/Byzantine [9,24]	31 (51.7%)	21 (35.0%)	4 (6.7%)	4 (6.7%)	60
c.898G>T (p.Ala300Ser)	Missense (Mod/Severe)	Euro-Mediterranean [1]	25 (52.1%)	15 (31.2%)	3 (6.3%)	5 (10.4%)	48
c.781C>T (p.Arg262Ter)	Nonsense (Null)	Western European [1,7]	15 (48.4%)	9 (29.0%)	6 (19.4%)	1 (3.2%)	31
c.143T>C (p.Leu48Ser)	Missense (Mild)	Indigenous/Italo-Greek [1]	13 (48.1%)	8 (29.6%)	3 (11.1%)	3 (11.1%)	27
c.1222C>T (p.Arg408Trp)	Missense (Null)	Northern European/Norman-Swabian [8,11]	13 (81.2%)	3 (18.8%)	0 (0.0%)	0 (0.0%)	16
c.441+5G>T (p.?)	Splicing (Null)	Euro-Mediterranean [11]	4 (25.0%)	10 (62.5%)	1 (6.3%)	1 (6.3%)	16

## Data Availability

The data presented in this study are available on request from the corresponding author.

## Abbreviations

ACMG	American College of Medical Genetics
AMP	Association for Molecular Pathology
ANOVA	Analysis of Variance
BA	Bari
BAT	Barletta-Andria-Trani
BH4	Tetrahydrobiopterin
BMI	Body Mass Index
BR	Brindisi
CI	Confidence Interval
FG	Foggia
HPA	Hyperphenylalaninemia
LE	Lecce
NGS	Next-Generation Sequencing
OR	Odds Ratio
PAH	Phenylalanine Hydroxylase
Phe	Phenylalanine
PKU	Phenylketonuria
PZ	Potenza
SD	Standard Deviation
SPSS	Statistical Package for the Social Sciences
TA	Taranto
